# Knowledge and determinants of women’s knowledge on vertical transmission of HIV and AIDS in South Africa

**DOI:** 10.1186/s12981-021-00367-7

**Published:** 2021-07-15

**Authors:** Eugene Kofuor Maafo Darteh, Susanna Aba Abraham, Abdul-Aziz Seidu, Vijay Kumar Chattu, Sanni Yaya

**Affiliations:** 1 Department of Population and Health, University of Cape Coast, Cape Coast, Ghana; 2 Department of Adult Health, School of Nursing and Midwifery, University of Cape Coast, Cape Coast, Ghana; 3 College of Public Health, Medical and Veterinary Sciences, James Cook University, Townsville, QLD Australia; 4 Department of Estate Management, Takoradi Technical University, Takoradi, Ghana; 5 Division of Occupational Medicine, Department of Medicine, University of Toronto, University of Toronto, Toronto, ON CanadaON Canada; 6 School of International Development and Global Studies, University of Ottawa, Ottawa, Canada; 7 The George Institute for Global Health, Imperial College London, London, UK; 8 Department of Public Health, Saveetha Institute of Medical and Technical Sciences, Saveetha University, Chennai, India

**Keywords:** Knowledge, PMTCT, HIV/AIDS, Women, South Africa, Public Health

## Abstract

**Background:**

HIV/AIDS is still one of the major public health concerns globally. It is one of the major contributory causes of deaths among women in the reproductive age (15–49 years) and has resulted in about 14 million orphaned children globally. Knowledge of Mother-to Child transmission is one of the strategies to fight against HIV. This study, therefore, sought to assess the knowledge and determinants of women’s knowledge on vertical transmission of HIV and AIDS in their reproductive age in South Africa.

**Methods:**

Data were obtained from the South Africa Demographic and Health Survey (SADHS) 2016. Both descriptive (frequencies and percentages) and inferential analysis (multilevel mixed-effects complementary log–log regression model) were conducted and the statistical significance was set at p < 0.05.

**Results:**

The prevalence of knowledge of mother to child transmission of HIV and AIDS during pregnancy, delivery, breastfeeding and at least knowledge of one source are 87.0%, 81.1%, 80.3% and 91.4% respectively. At the individual level, those with secondary [AOR = 1.28, CI = 1.04,1.57] and higher [AOR = 1.55, CI = 1.21,1.99], those who read newspaper less than once a week [AOR = 1.16, CI = [1.05,1.28], at least once a week [AOR = 1.14, CI = 1.04,1.25], and those who listen to the radio less than once a week [AOR = 1.22, CI = 1.03,1.43] had higher odds of knowledge on MTCT of HIV and AIDS. However, those with parity 0 [AOR = 0.73, CI = [0.63,0.85] had lower odds of knowledge of MTCT of HIV and AIDS compared with those with parity 4 or more. At the contextual level, those in the poorest wealth quintile [AOR = 0.82,CI = 0.69,0.97] had lower odds of having knowledge of MTCT of HIV and AIDS. Those in the urban areas [AOR = 1.17, CI = [1.04,1.31], those in Limpopo [AOR = 1.35, CI = [1.12,1.64], Gauteng [AOR = 1.35, CI = [1.12,1.62] and North west[AOR = 1.49, CI = [1.22,1.81] had higher odds of knowledge of mother to child transmission of HIV and AIDS.

**Conclusion:**

The study has demonstrated that there is relatively high knowledge of mother to child transmission of HIV and AIDS in South Africa. The factors associated with the knowledge are educational level, exposure to mass media, parity, wealth status, place of residence and the region of residence. To further increase the knowledge, it is imperative to adopt various messages and target respondents in different part of SSA through the mass media channels. This should be done taking cognizant of the rural–urban variations and socio-economic status.

## Introduction

Since the onset of the HIV epidemic, AIDS is the primary cause of mortality among women in the reproductive age (15–49) and has resulted in about 14 million orphaned children globally [1]. An estimated 150 000 children became newly infected with HIV in 2019 only [2]. South Africa has a generalized epidemic and has an estimated 7.5 million persons living with HIV (PLHIV), the highest worldwide [3]. Similar to other global trends, about 4.7 million of the PLHIV are women and 69, 000 of these infections occurred in women aged 15–24 in 2018 [4]. An estimated 460,000 children were infected in 2018 [5] and mother-to-child transmission (MTCT) continuous to be the leading cause of HIV infection in younger children, especially those under-five years.

Without any interventions, HIV exposed children are at 20% to 40% risk of being infected with the virus [6]. Hence, in 2016, the World Health Organization (WHO) endorsed strategies that will virtually eliminate MTCT by achieving zero new HIV infections in infants by 2020 [7]. Although significant strides have been gained in reducing MTCT in South Africa, HIV continuous to be a leading cause of maternal and under-five deaths [8]. WHO outlined a four-pronged approach to eliminate paediatric HIV [7]. The approach seeks to prevent HIV infection among women in the reproductive age; preventing unintended pregnancy among women living with HIV; preventing HIV transmission from a woman living with HIV to her infants; and providing appropriate treatment, care and support to mothers living with HIV, their children and families [7]. Much efforts have been made in establishing the infrastructure and programs for providing the services in the health system [8]. However, the women who will utilize the service are a critical component for achieving the goal of virtual elimination of vertical transmission.

Several social and behavioural change communication programmes have been rolled-out in South Africa to improve accurate knowledge on HIV, MTCT and PMTCT among women in the fertility age [9, 10]. However, studies based on the South Africa’s 2012 and 2017 National HIV, Behaviour and Health Survey have reported that accurate knowledge of HIV transmission and prevention among respondents including women in the reproductive ages have generally remained poor [11, 12]. Accurate MTCT and PMTCT knowledge among women in the reproductive age is essential as it impacts behaviour change and promotes adoption of self-preserving attitudes such as increased perceived vulnerability, condom use and HIV testing [13]. There is therefore the need to formulate policies that promote accurate knowledge sharing and awareness of HIV issues among the general population especially women in their reproductive age [14]. We therefore aimed to determine the level of knowledge of South African women in the reproductive age on the modes of transmission of HIV from mother-to-child and to determine the predictors of the comprehensive MTCT knowledge. The findings will shed light on the prevalence indices that could inform future policies on improving women’s knowledge on PMTCT and other reproduction-related subjects in South Africa.

## Materials and methods

### Source of data

The current study is a cross-sectional analysis of nationally representative data from the 2016 South African Demographic and Health Survey (SADHS). The primary purpose of the DHS was to provide up-to-date estimates of basic demographic and health indicators which include fertility levels, maternal and childhood mortality, immunization coverage, HIV testing and counselling, and physical and sexual violence against women. Another objective was to provide estimates of health and behaviour indicators in adults aged 15 and older.

The SADHS 2016 followed a stratified two-stage sampling design with a probability proportional to size sampling of PSUs at the first stage, and systematic sampling of residential dwelling units (DUs) at the second stage. Each province was stratified into an urban, farm, and traditional areas, yielding 26 sampling strata, from which 750 PSUs were selected. DUs within each PSU were listed, and this list served as a frame for sampling DUs. Data collection for the SADHS 2016 took place from 27 June 2016 to 4 November 2016. Data were collected using questionnaires administered by conducting face-to-face interviews. Details of the questionnaires sampling and data collection procedure have been published in the final report [15]. Thus, a dataset was created from information obtained from these questionnaires. From the dataset, we included 7861 all women aged 15–49 who had complete information on all the variables of interest constituted our sample.

## Study variables

### Outcome variables

The main outcome variable for this study was knowledge of MTCT of HIV. Three main questions on the transmission of HIV from mother to child during pregnancy, delivery and breastfeeding were used to assess MTCT knowledge. Each of these questions had three responses: Yes, No, and Don’t Know. Based on previous studies [16, 17], No and Don’t know were treated as No = 0 and Yes = 1. Afterwards, an index was generated for all the “yes” and “no” responses, with scores ranging from 0 to 3. A score of 0 was labelled as “No”, and scores 1 to 3 as “Yes”.

## Independent variables

In this study, we had both individual and contextual level factors that influence women's knowledge of MTCT. At the individual level, age (15–19, 20–24, 25–29, 30–34, 35–39, 40–44, 45–49), employment (not working, managerial, clerical, Agriculture, Home, Services, manual), marital status (Never married, married, Cohabiting, Widowed/Divorced/Separated), education (no education, primary, secondary, Higher), exposure to mass media (Radio, TV and Newspaper) (Not at all, Less than once a week, At least once a week) and parity (0, 1, 2, 3, 4 +) were considered. At the contextual level, wealth status (poorest, poorer, middle, Richer, Richest), Region (Western Cape, Eastern Cape, Northern Cape, Free State, KwaZulu-Natal, Northwest, Gauteng, Mpumalanga, Limpopo), type of residence (urban, rural) was considered. These variables were not just considered arbitrarily, but based on previous studies in sub-Saharan Africa [18–20] including South Africa [21–23].

### Statistical analyses

The data were analyzed with Stata version 14.2 for Mac OS. The analysis was done in three steps where descriptive analysis of the background characteristics was done initially, followed by calculating the prevalence and proportions of knowledge of mother to child transmission of HIV and AIDS across the socio-demographic characteristics through a cross-tabulation (see Table 1). At the inferential level, a two-level multilevel mixed-effects complementary logistic regression models were built to accommodate the stratified multistage sampling technique employed in the SDHS. This means that the women were nested within households and households were nested within clusters. The multilevel mixed-effects models allow assessment of the effect of hierarchical ordering on the variances of the predictor variables of the analysis. Because the outcome variable was not evenly distributed, we employed the complementary logistic regression function instead of the standard binary logistic regression function. The dependent variable in this study was binary, however its distribution was uneven. This indicates that the outcome distribution violates the symmetry assumption of a normal binary logistic regression model. The multilevel mixed-effects complementary log–log regression model relaxes the binary logistic regression model's symmetry assumption, ensuring that parameter estimations for events with asymmetrical distribution are not biased ([24], p.8). We considered clusters as random effects to take care of unexplained variability at the contextual level. Specifically, three models were built. The first model (Model 0) had no predictors. The second model (Model 1) had only the individual level factors. Model 2 had only the contextual level factors whereas the final model, Model 3 had both individual and contextual level factors. The multilevel mixed-effects complementary log regression model had both fixed and random effects. At the fixed effects results section, we presented the results as adjusted odds ratio (aOR) alongside their corresponding 95% confidence intervals (CI). At the random effects section, we used the intra-cluster correlation (ICC) to present the results. Log-likelihood ratio (LLR) and Akaike’s Information Criterion (AIC) tests were used to present model comparisons. Multicollinearity was checked using the variance inflation factor, and there was no evidence of multicollinearity among the variables (Mean VIF = 1.25, Maximum VIF = 1.50, Minimum VIF = 1.02). All frequency distributions were weighted while the survey command (svy) in Stata was used to adjust for the complex sampling structure of the data in the regression analyses.

**Table 1:** Background characteristics and prevalence of knowledge of mother to child transmission of HIV and AIDS

	Weighted sample		During pregnancy (%)	During delivery (%)	During breastfeeding (%)	At least one (%)
Prevalence			87.01	81.10	80.3	91.4
Age	n	%				
15–19	1343	17.1	80.68	72.6	85.1	85.08
20–24	1312	16.7	87.13	81.2	92.0	92.03
25–29	1337	17.0	89.82	82.8	93.8	93.79
30–34	1221	15.5	89.92	84.0	94.8	94.79
35–39	986	12.5	86.18	81.2	91.4	91.39
40–44	850	10.8	88.01	80.7	91.1	91.12
45–49	813	10.3	88.21	79.9	92.2	92.17
Employment						
Not working	4943	62.9	85.93	79.46	79.79	90.08
Managerial	625	8.0	92.35	86.20	81.12	96.73
Clerical	510	6.5	87.06	84.95	79.21	92.67
Agricultural	88	1.1	92.68	89.86	88.20	96.51
Home	386	4.9	86.57	81.11	82.29	92.03
Services	579	7.4	86.93	81.33	79.15	92.85
Manual	730	9.3	89.26	83.92	82.25	92.88
Marital status						
Never married	4600	58.5	85.60	78.77	79.46	90.20
Married	1830	23.3	89.17	85.12	81.64	93.59
Cohabitation	995	12.7	88.91	83.24	81.60	91.88
Widow/Divorced/Separated	435	5.5	88.39	83.88	79.71	93.85
Education						
No education	152	1.9	81.03	74.19	75.06	85.88
Primary	704	9.0	83.08	77.24	77.57	86.21
Secondary	6072	77.2	87.04	80.65	80.43	91.46
Higher	934	11.9	90.74	88.02	81.98	95.87
Wealth						
Poorest	1523	19.4	83.19	76.52	78.67	87.56
Poorer	1569	20.0	86.15	79.68	81.08	90.85
Middle	1666	21.2	85.61	80.99	79.49	90.65
Richer	1660	21.1	89.88	84.14	82.22	93.15
Richest	1444	18.4	90.27	84.11	79.65	94.94
Parity						
0	2197	28.0	82.52	72.15	75.08	87.67
1	1902	24.2	89.32	83.42	82.30	93.23
2	1825	23.2	89.67	86.93	83.65	94.07
3	1092	13.9	87.46	83.09	80.99	91.61
4 and above	845	10.8	87.13	83.97	80.80	90.99
Region						
Western cape	903	11.5	86.33	78.27	65.12	92.02
Eastern Cape	907	11.5	77.92	71.17	73.23	85.62
Northern Cape	155	2.0	82.71	71.89	78.01	89.02
Free state	420	5.3	87.85	81.65	81.06	90.97
Kwazulu-Natal	1484	18.9	88.80	87.05	84.97	91.22
Northwest	551	7.0	91.24	79.16	80.52	95.55
Gauteng	2078	26.4	92.00	88.68	88.64	95.04
Mpumalanga	633	8.1	79.25	70.05	71.25	84.12
Limpopo	731	9.3	85.25	75.96	81.84	91.80
Residence						
Urban	5295	67.4	89.16	84.01	81.71	93.22
Rural	2566	32.6	82.56	75.10	77.24	87.66
Reading newspaper/magazine						
Not at all	2772	35.3	83.78	77.16	78.68	88.16
Less than once a week	1952	24.8	88.91	82.71	81.76	92.89
At least once a week	3137	39.9	88.68	83.57	80.71	93.35
Frequency of watching TV						
Not at all	2321	29.5	84.96	78.41	78.56	88.91
Less than once a week	1224	15.6	87.81	81.78	81.38	92.10
At least once a week	4317	54.9	87.88	82.35	80.85	92.55
Frequency of listening to radio						
Not at all	1331	16.9	83.92	80.67	81.37	88.29
Less than once a week	732	9.3	91.83	84.93	83.42	94.86
At least once a week	5798	73.8	87.11	80.71	79.60	91.68

### Ethical consideration

This study was based on analyses of secondary data set from the DHS program, which gave us permission for its use. The survey was approved by the Institutional Review Board (IRB) of ICF Macro International in the United States and the National Ethics Committee in the Federal Ministry of Health of South Africa. All participants in the survey gave their consent to participate.

## Results

### Background characteristics and prevalence of knowledge of mother to child transmission of HIV and AIDS

Table 1 presents the background characteristics of the study and the prevalence of knowledge of MTCT of HIV and AIDS. Seventeen percent (17.1%) of the respondents were aged 15–19, 62.9% were not working, 58.5% were never married, and 77.2% had secondary level of education. The study also showed that 21.2% are in the middle wealth quintile, 26.4% were from Gauteng region, and 67.4% are in urban centres. The results further indicate that 39.9%, 54.9% and 73.8% are respectively exposed to newspaper, radio and TV at least once a week. The prevalence of knowledge of mother to child transmission of HIV and AIDS during pregnancy, delivery, breastfeeding and at least one knowledge is 87.0%, 81.1%, 80.3% and 91.4% respectively (Table 1).

### Fixed effects (measure of association) results

Table 2 shows the individual and contextual factors associated with knowledge of MTCT of HIV and AIDS. At the individual level, with education, it was found that those with secondary [AOR = 1.28, CI = 1.04,1.57] and higher [AOR = 1.55, CI = 1.21,1.99] levels of education had higher odds of knowledge of MTC of HIV and AIDS compared with those with no formal education. With media exposure, the study further shows that those who read newspaper less than once a week [AOR = 1.16, CI = [1.05, 1.28], at least once a week [AOR = 1.14, CI = 1.04, 1.25], and those who listen to the radio less than once a week [AOR = 1.22, CI = 1.03,1.43] had higher odds of knowledge on MTCT of HIV and AIDS compared with those who are not exposed at all. However, those with parity 0 [AOR = 0.73, CI = [0.63, 0.85] had lower odds of knowledge of MTCT of HIV and AIDS compared with those with parity 4 or more. At the contextual level, those in the poorest wealth quintile [AOR = 0.82,CI = 0.69 ,0.97] had lower odds of having knowledge of MTCT of HIV and AIDS compared with those in the richest wealth quintile. Those in the urban areas[AOR = 1.17, CI = [1.04, 1.31] had higher odds of having knowledge on MTCT of HIV and AIDS compared with those in rural areas. With a region of residence, those in Limpopo [AOR = 1.35, CI = [1.12, 1.64], Gauteng [AOR = 1.35, CI = [1.12, 1.62] and North west[AOR = 1.49, CI = [1.22, 1.81] had higher odds of knowledge of mother to child transmission of HIV and AIDS compared with those in Northern cape (Table 2).

**Table 2:** Multilevel mixed-effects complementary log–log regression model showing the association between individual and contextual factors on the knowledge of mother to child transmission of HIV and AIDS

Variables	Model 0	Model 1	Model 2	Model 3
		AOR [95%CI]	AOR [95%CI]	AOR [95%CI]
Individual level				
Age				
15–19		0.89 [0.75,1.06]		0.92 [0.77,1.09]
20–24		1.11 [0.95,1.31]		1.15 [0.98,1.35]
25–29		1.05 [0.91,1.22]		1.09 [0.94,1.26]
30–34		1.07 [0.92,1.24]		1.11 [0.96,1.28]
35–39		0.95 [0.83,1.10]		0.96 [0.83,1.11]
40–44		0.95 [0.82,1.10]		0.95 [0.82,1.09]
45–49		Ref		Ref
Employment				
Not working		0.84 [0.58,1.22]		0.83 [0.58,1.20]
Managerial		0.95 [0.63,1.43]		0.93 [0.63,1.38]
Clerical		0.78 [0.52,1.17]		0.76 [0.51,1.13]
Agricultural		Ref		Ref
Home		0.87 [0.58,1.30]		0.85 [0.57,1.26]
Services		0.91 [0.61,1.35]		0.87 [0.60,1.28]
Manual		0.92 [0.62,1.35]		0.90 [0.62,1.32]
Marital status				
Never married		0.94 [0.80,1.10]		0.93 [0.79,1.08]
Married		0.93 [0.79,1.10]		0.92 [0.78,1.08]
Cohabitation		0.97 [0.80,1.16]		0.95 [0.79,1.14]
Widow/Divorced/Separated		Ref		Ref
Education				
No education		Ref		Ref
Primary		1.08 [0.87,1.33]		1.09 [0.88,1.34]
Secondary		1.29* [1.05,1.59]		1.28* [1.04,1.57]
Higher		1.65*** [1.28,2.11]		1.55*** [1.21,1.99]
Parity				
0		0.77*** [0.66,0.90]		0.73*** [0.63,0.85]
1		0.90 [0.79,1.04]		0.88 [0.77,1.01]
2		0.97 [0.85,1.11]		0.95 [0.83,1.08]
3		0.98 [0.85,1.12]		0.95 [0.83,1.09]
4 or more		Ref		
Frequency of reading Newspaper				
Not at all		Ref		Ref
Less than once a week		1.16** [1.05,1.27]		1.16** [1.05,1.28]
At least once a week		1.17*** [1.06,1.28]		1.14** [1.04,1.25]
Frequency of watching TV				
Not at all		Ref		Ref
Less than once a week		1.06 [0.95,1.18]		1.05 [0.94,1.17]
At least once a week		1.10* [1.01,1.19]		1.08 [0.99,1.17]
Frequency of listening to radio				
Not at all		Ref		Ref
Less than once a week		1.29** [1.10,1.52]		1.22* [1.03,1.43]
At least once a week		1.06 [0.95,1.17]		0.982 [0.88,1.10]
4 and above		Ref		Ref
Contextual factors				
Region				
Northern Cape			Ref	Ref
Western Cape			1.04 [0.87,1.24]	1.02 [0.85,1.22]
Eastern Cape			0.94 [0.80,1.11]	0.93 [0.78,1.10]
Free State			1.04 [0.88,1.23]	1.04 [0.88,1.24]
KwaZulu-Natal			1.15 [0.94,1.39]	1.13 [0.92,1.38]
North West			1.52*** [1.25,1.84]	1.49*** [1.22,1.81]
Gauteng			1.36*** [1.13,1.63]	1.35** [1.12,1.62]
Mpumalanga			0.91 [0.76,1.08]	0.89 [0.75,1.06]
Limpopo			1.32** [1.10,1.58]	1.35** [1.12,1.64]
Residence				
Urban			1.17** [1.05,1.30]	1.17** [1.04,1.31]
Rural			Ref	Ref
Wealth				
Poorest			0.75*** [0.65,0.87]	0.82* [0.69,0.97]
Poorer			0.84* [0.73,0.97]	0.87 [0.75,1.01]
Middle			0.87 [0.77,1.00]	0.89 [0.77,1.03]
Richer			0.94 [0.83,1.06]	0.97 [0.85,1.10]
Richest			Ref	Ref
Random effects results				
PSU variance	0.15	0.15	0.10	0.11
ICC	0.20	0.15	0.15	0.15
Wald chi-square and p-value	Ref	160.68 (p < 0.001)	113.60 (p < 0.001)	248.83 (p < 0.001)
LR Test	176.72 (p < 0.001)	152.57 (p < 0.001)	92.78 (p < 0.001)	93.02 (p < 0.001)
Model fitness				
Log-likelihood	− 2453.69	− 2359.58	− 2401.68	− 2319.44
AIC	4911.38	4779.151	4833.369	4724.882
PSU	723	723	723	723
*N*	7861	7861	7861	7861

Exponentiated coefficients; 95% confidence intervals in brackets

^*^p < 0.05, ^**^p < 0.01, ^***^p < 0.001, *Ref*   Reference category, *PSU*  Primary sampling unit, *ICC * Intra-class correlation, *LR* Test  Likelihood ratio test, *AIC   *Akaike’s information criterion

### Random effects (measures of variations) results

The empty model (Model 0), as shown below in Table 2, depicted a substantial variation in the likelihood knowledge of mothers on MTCT in south Africa across the Primary Sampling Units (PSUs) clustering [σ2 = 15%). The Model 0 indicated that 20% of the variation in MTCT of HIV was attributed to Intra-Class Correlation variation, i.e., (ICC = 0.20). The variation between-cluster decreased to 15% (0.15) in the remaining models. The Akaike’s Information Criterion (AIC) values showed a reduction, which means a substantial improvement in each of the models over the previous model and also affirmed the goodness of Model III developed in the analysis. Therefore, Model III, the complete model with both the selected individual and household/community factors, was selected as the best model to explain the factors associated with mothers’ knowledge on mother to child transmission of HIV and AIDS.

## Discussion

South Africa is one of the countries with high HIV prevalence globally [25]. This disproportionally affect more women and the possibility of these women passing it on to their unborn children are very high [21]. Evidence also reveal that, HIV prevalence is low among people with high knowledge of HIV [26]. Based on this, it is prudent to assess the associative effects of demographic factors on the knowledge of HIV transmission from mother to children. This study, therefore, sought to assess the prevalence and determinants of knowledge on MTC of HIV and AIDS among women in South Africa. The prevalence of knowledge of mother to child transmission of HIV and AIDS during pregnancy, delivery, breastfeeding and at least one source of knowledge is 87.0%, 81.1%, 80.3% and 91.4% respectively. The study also found that education, wealth status, parity, region of residence, place of residence, and exposure to radio and newspaper are associated with knowledge on MTCT of HIV and AIDS. The prevalence on the knowledge of MTCT of HIV and AIDS is similar to what has been observed in Ethiopia by Malaju, and Alene [27] but higher than what was found to be 60% in Nigeria [28], and 50% in Tanzania [29] and more than 60% in Uganda [30].

The study found that those in high socio-economic status (high education and wealth) had higher odds of knowing MTCT of HIV and AIDS. This is similar to several previous studies [18, 19, 27, 31, 32]. The plausible explanations to this finding can be viewed in several pathways. For example, educated women might be able to easily comprehend the health education they receive compared to uneducated women [31]. Also, previous studies have espoused that higher socio-economic status is associated with easy access to healthcare including health information [32–35]. Furthermore, women who have attained higher education are more likely to secure jobs with high wages and are able to afford various means to access health information [32].

Similar to previous studies [18, 20, 31, 36], place of residence and regional variations were observed in the knowledge of women on MTCT of HIV and AIDS. Women in urban areas are more likely to have knowledge compared to those in rural areas. This perhaps is due to the inter-regional and rural–urban differentials in access to education and resources including HIV and AIDS education [18, 20]. Liyeh et al. [18] also noted that women in urban centres there is easy accessibility and availability of nearby health services and greater media exposure compared with rural areas.

Relatedly, the study also showed that exposure to mass media (radio and newspaper) were associated with a higher odd of MTCT of HIV and AIDS knowledge. This corroborates several previous studies [19, 32, 37, 38]. Mass media, especially, radio is a useful tool to convey health education messages in different languages to the intended audience [20]. This can, therefore, be a valuable channel to tackle those with a low level of education [19, 31].

Besides, it was observed that those with parity 0 had lower odds of knowledge of MTC of HIV and AIDS. The possible explanation to this is that women who had given birth before and attended either ANC or PNC are more likely to benefit from the health education the health providers give during these services Yaya et al. [26] compared to those who have never accessed these services before. This is consistent with previous findings that ANC attendance is associated with higher odds of knowledge on mother to child transmission of HIV and AIDS [18]. Yaya et al. [26] discussed that there is the possibility for women to attain information on HIV transmission as well as the predisposing factors. By this they can also seek professional care during ANC to prevent vertical transmission.

## Strength and limitations

The major strength of this study is the use of the most recent nationally representative DHS data. The sample size was relatively large, that allowed the use of rigorous statistical analysis. Despite this, the DHS adopts a cross-sectional design which therefore precludes causal interpretations of the results. Finally, there is also the possibility of social desirability biases from the respondents.

## Conclusion

The study has demonstrated that there is relatively high knowledge of mother to child transmission of HIV in South Africa. The determinants are educational level, wealth status, place of residence, the region of residence, exposure to mass media and parity. Despite this, there were variations in the factors associated with it. To further increase the knowledge, it is imperative to adopt various messages and target respondents in various part of South Africa through the mass media channels especially radio. This should be done taking cognizance of the rural–urban variations and socio-economic differentials.

## Data Availability

Data is available on https://dhsprogram.com/data/dataset/South-Africa_Standard-DHS_2016.cfm?flag=0.

## Abbreviations

AIC: Akaike’s information criterion
AOR: Adjusted odds ratio
CI: Confidence interval
ICC: Intra-class correlation
LR Test: Likelihood ratio test
MTCT: Mother-to-child transmission
PSU: Primary sampling unit
SADHS: South Africa Demographic and Health Survey
SDG: Sustainable development goal
SSA: Sub-Saharan Africa
Stats SA: Statistics South Africa
UNGASS: United Nations General Assembly Special Session on Drugs
VIF: Variance inflation factor
WHO: World Health Organisation
